# Emerging trends in chiral inorganic nanomaterials for enantioselective catalysis

**DOI:** 10.1038/s41467-024-47657-y

**Published:** 2024-04-25

**Authors:** Si Li, Xinxin Xu, Liguang Xu, Hengwei Lin, Hua Kuang, Chuanlai Xu

**Affiliations:** 1 The Key Laboratory of Synthetic and Biological Colloids, Ministry of Education, School of Chemical and Material Engineering, Jiangnan University, Wuxi, Jiangsu 214122 China; 2 International Joint Research Laboratory for Biointerface and Biodetection, Jiangnan University, Wuxi, Jiangsu 214122 People’s Republic of China; 3 State Key Laboratory of Food Science and Technology, Jiangnan University, Wuxi, Jiangsu People’s Republic of China

**Keywords:** Heterogeneous catalysis, Catalyst synthesis, Nanoparticle synthesis

## Abstract

Asymmetric transformations and synthesis have garnered considerable interest in recent decades due to the extensive need for chiral organic compounds in biomedical, agrochemical, chemical, and food industries. The field of chiral inorganic catalysts, garnering considerable interest for its contributions to asymmetric organic transformations, has witnessed remarkable advancements and emerged as a highly innovative research area. Here, we review the latest developments in this dynamic and emerging field to comprehensively understand the advances in chiral inorganic nanocatalysts and stimulate further progress in asymmetric catalysis.

## Abstract (web summary)

Considerable attention has been directed towards chiral nanocatalysts due to their significant role in facilitating asymmetric organic transformations. Here the authors highlight the recent advancements and notable examples in the field of chiral inorganic nanocatalysts.

## Introduction

Chiral catalysis has emerged as one of the most effective approaches for obtaining chiral organic compounds, allowing for a remarkable acceleration of chemical reactions up to 10^19^ times^1^. Chiral catalysts play pivotal roles in enhancing the reaction rates of chemical organic synthesis^2–7^. Over the past two decades, chiral nanocatalyst-mediated enantioselective organic synthesis and transformation have achieved considerable attention and become a significant area of research^8^.

Significant progress has been made in pursuing high-performing chiral catalysts, resulting in new, versatile, and efficient methods for developing chiral catalysts. The current chiral catalysts are primarily constructed using chiral ligands, chiral metal complexes, biological catalysts, and small organic molecules^9–13^. Among these catalysts, chiral inorganic nanocatalysts have garnered increasing attention in recent years^14^.

**Fig. 1:**
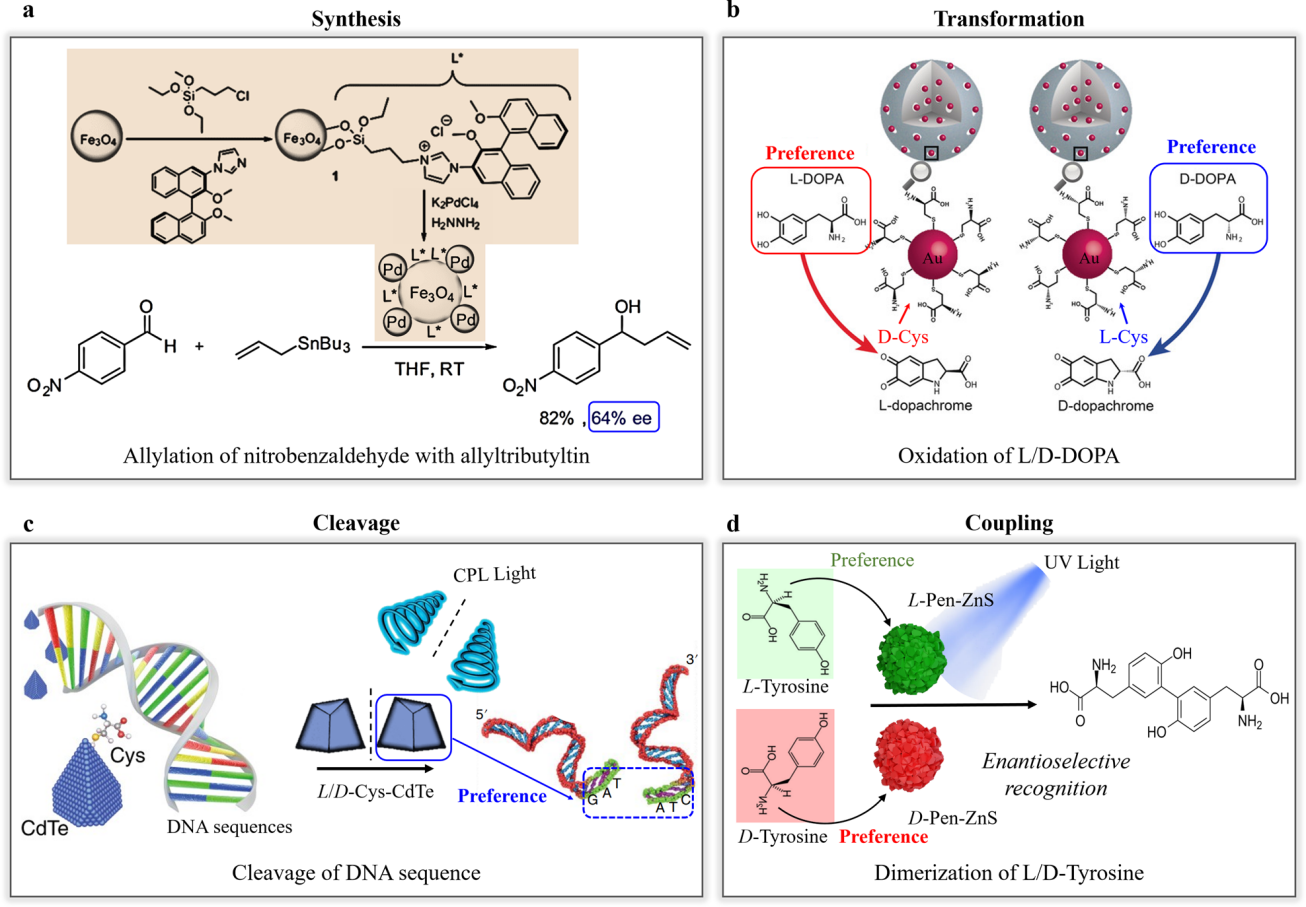
Chiral inorganic nanocatalysts mediated enantioselective reactions. **a** Enantioselective chiral synthesis^34^. **b** Enantioselective transformation of chiral molecules^36^. **c** Enantioselective cleavage of chiral macromolecules^37^. **d** Enantioselective coupling between chiral molecules^38^.

Chiral inorganic nanomaterials can be synthesized not only with enantioselective catalytic performances like natural enzymes^9,12,14–30^, but also with significant desirable properties, including recyclability, convenience in construction and storage, catalytic efficiency, structural stability, and economic performance. These properties can be obtained by optimizing the synthetic pathway, selecting proper compositions, utilizing computational prediction assistance, and precisely optimizing the structure^31–33^. They are widely applied in chiral organic synthesis (Fig. 1a)^34^, enantioselective transformations of chiral molecules (Fig. 1b)^35^, enantioselective cleavages of chiral macromolecules (Fig. 1c)^36^, and enantioselective coupling of chiral small molecules^37,38^ (Fig. 1d). To comprehensively understand these advances, this article introduces the latest progress and representative work on chiral inorganic nanocatalysts.

## Chiral inorganic nanocatalysts

Chiral inorganic nanocatalysts are mainly synthesized by serval main methods^12,13,17,39,40^. Firstly, enantioselective catalysts are developed through the combination of achiral inorganic nanoparticles (NPs) with chiral organic catalysts^41^ or by linking achiral organic catalysts with chiral fragments^42^. The catalytic and enantioselective recognition centers are attributed to the organic part, while the inorganic nanomaterials only serve as supports. Secondly, inorganic nanomaterials act as both supports and catalytic centers. Enantioselective recognition centers are established by modifying the surface of these nanomaterials with chiral ligands. Thirdly, inorganic nanomaterials can function as both catalytic centers and enantioselective centers. Typically, the chiral recognition center of chiral inorganic nanocatalysts is created by encoding chiral information within the inorganic nanomaterials themselves with the assistance of chiral molecules.

It is widely recognized that the chemical compositions and physical morphologies of chiral inorganic nanocatalysts strongly influenced their catalytic and enantioselective performances. Except for the construction methods mentioned above, optimization techniques also involve meticulous composition selection, controllable structure adjustments, precise design of chiral recognition spaces, and simplification of synthetic methods. To facilitate a clear understanding of chiral inorganic nanocatalysts’ advancements, they are categorized and introduced based on their inorganic compositions.

### Chiral metal nanocatalysts

**Fig. 2:**
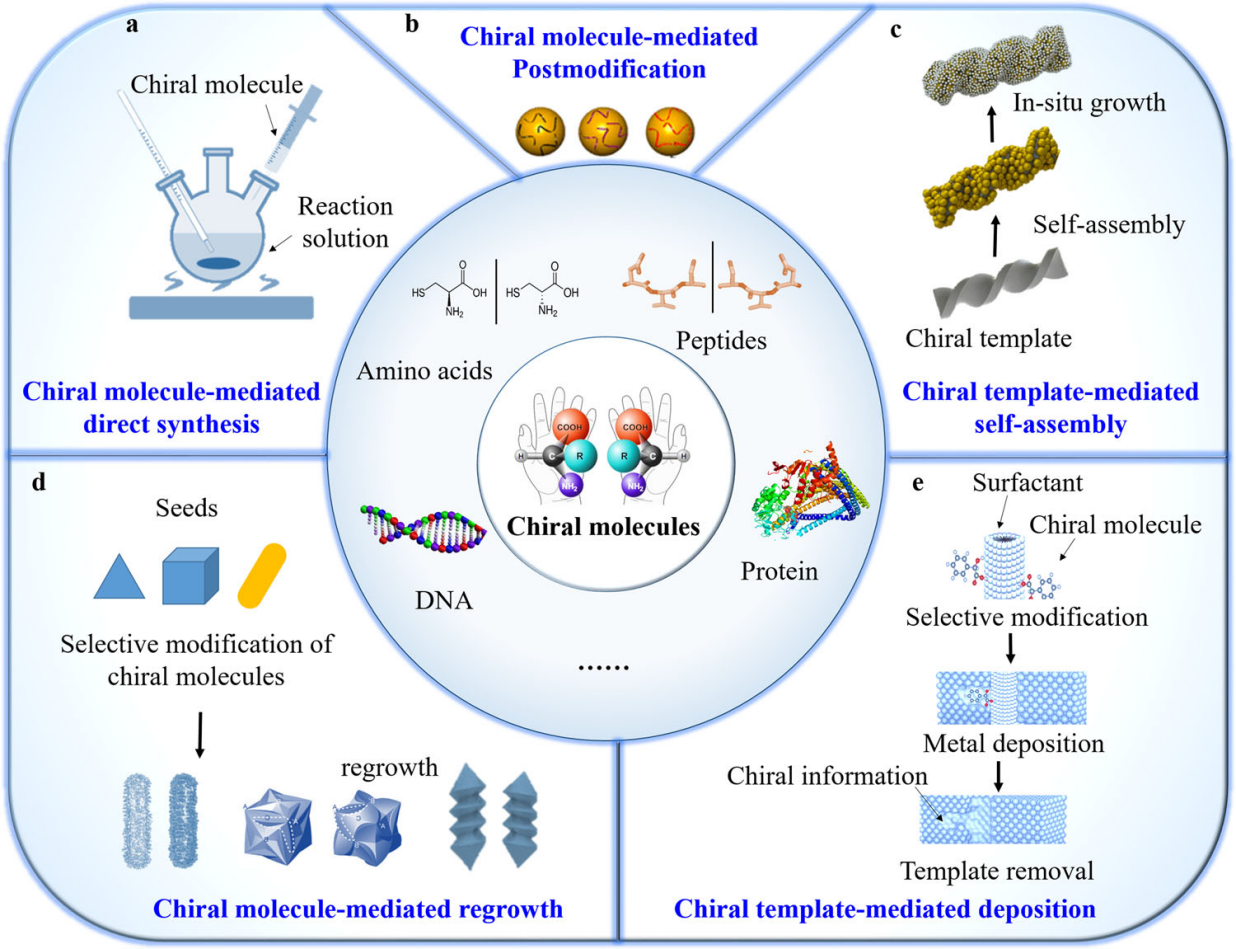
Construction methods of chiral inorganic nanomaterials. **a** Chiral molecules-mediated direct synthesis^38,39,48^, **b** chiral molecules-mediated post-modification (Copyright © 2015, American Chemical Society)^50,51^, **c** chiral molecules-mediated regrowth of metal seeds (Copyright © 2022, American Chemical Society)^52^, **d** chiral templates mediated in situ growth or self-assembly of metal materials^22^, **e** chiral molecules-mediated in situ deposition of metal (Copyright © 2014, The Authors)^53,54^.

In contrast to macro-scale metals, metal materials at the nanoscale can be finely engineered to exhibit specific physical, optical, and catalytic properties^8,25,43–46^. This capability has gained significant momentum and finds wide applications in chemical industries, petroleum refining, pharmaceuticals, environmental sustainability, and new energy technologies as catalysts. Chiral molecules, such as amino acids, peptides, proteins, and DNA sequences, play indispensable roles in constructing chiral metal nanocatalysts. The typical construction methods include chiral molecule-guided direct synthesis (Fig. 2a)^37,38,47,48^, chiral molecule-mediated post modification on the surface of metal nanomaterials (Fig. 2b)^49,50^, chiral templates-mediated in situ growth of metal materials or assembly with metal nanomaterials (Fig. 2c)^51^, chiral molecules mediated regrowth of metal seeds (Fig. 2d)^22^, and chiral template-mediated deposition (Fig. 2e)^52,53^. The constructed chiral metal nanocatalysts exhibit unique physiochemical properties through meticulous control of synthetic methods and parameters^54–57^.

Initially, chiral metal nanocatalysts were typically created by surface modification of synthesized metal nanomaterials using chiral molecules. For instance, thiols containing chiral Zn^2+^-binding head groups were assembled onto the surface of Au NPs. These modified NPs exhibited remarkable enantioselectivity and demonstrated RNA cleavage abilities towards dinucleotides such as UpU, GpG, ApA, and CpC^45^. Another example involves the synthesis of metal NPs functionalized with sugammadex, a carboxylic acid-functionalized γ-cyclodextrin derivative. These modified NPs exhibited a high chiral recognition ability towards lysine and asparagine enantiomers and were utilized in the catalytic reduction of toxic 4-nitrophenol mediated by NaBH_4_^58^. Furthermore, rhodium NPs modified with chiral diene exhibited catalytic activity in asymmetric 1,4-addition reactions upon activation by reductive reagents^44^.

Recently, there have been significant advancements in the development of chiral metal nanocatalysts, incorporating new features to enhance their enantioselective catalytic capabilities. Toste and Somorjai^59^ explored the in situ reduction of HAuCl_4_ to load gold nanoclusters onto mesoporous SiO_2_, resulting in the creation of chiral heterogeneous catalysts. These catalysts were developed by coating a chiral self-assembled monolayer on the internal surface of mesoporous silica. The chiral NPs inside the mesoporous structure acted as catalytic centers, while the silicon skeleton provided chiral space and sites. Remarkably, this chiral catalyst exhibited up to 50% enantioselectivity in forming cyclopropane-containing products. Qu and coworkers utilized L-/D-cysteine (L-/D-Cys) to modify Au NPs loaded in the mesoporous silicon NPs, resulting in high enantioselective transformation from L-/D-DOPA to L-/D-dopachrome (Fig. 1b)^35^.

DNA molecules can also be employed as chiral selectors in the design of chiral metal nanocatalysts. Ding et al.^50^ utilized environment-responsive DNA sequences to modify the surface of Au NPs for selective oxidation reactions. The DNA molecules modified on the surface of Au NPs can undergo conformational changes (such as switching between randomly coiled and multi-stranded structures like duplex, i-motif, or G-quadruplex) in response to pH stimuli. It was observed that the randomly coiled DNA-modified Au NPs exhibited a strong preference for L-glucose. In contrast, the structured DNA-modified Au NPs displayed higher catalytic activity towards D-glucose. This study exemplified the glucose oxidase-like catalytic behavior of DNA sequence-modified Au NPs, demonstrating the influence of chiral selectors on the enantioselective catalytic ability.

In addition to using enantiomeric molecules as chiral selectors, metal matrices can also achieve enantioselective recognition and catalysis by encoding chiral information of enantiomeric molecules into metal materials. In 2014, Kuhn and colleagues employed this approach^53^, electrochemically reducing platinum salts in the presence of chiral template molecules in a liquid phase, to create metal materials with multiple chiral recognition spaces. After removing the chiral molecules and liquid phases, mesoporous platinum electrodes were formed, showcasing effective enantiomer discrimination. Specifically, the mesoporous electrodes carrying the chiral information of L-DOPA exhibited enhanced electro-oxidation of L-DOPA compared to D-DOPA. In 2016, Kuhn’s team^60^ expanded on this concept and utilized chiral mesoporous platinum structures for the asymmetric electrochemical synthesis of mandelic acid. When electrodes imprinted with the (R)-enantiomer were used, a high enantiomeric excess of the (R)-enantiomer was observed, whereas the (S)-enantiomer was favored with (S)-imprinted electrodes. Although these monometallic matrices displayed chiral features and high enantioselective catalytic abilities, their practical applications were limited due to their low catalytic stability. In 2021, Kuhn’s group^52^ addressed this limitation by constructing mesoporous nanostructures alloyed with platinum and iridium using the same encoding method. These alloyed structures exhibited enhanced enantioselectivity in asymmetric electrosynthesis and demonstrated high electrochemical catalytic stability. This chiral inorganic nanocatalyst construction method could solve the dimensional mismatch between catalysts and substrates.

**Fig. 3:**
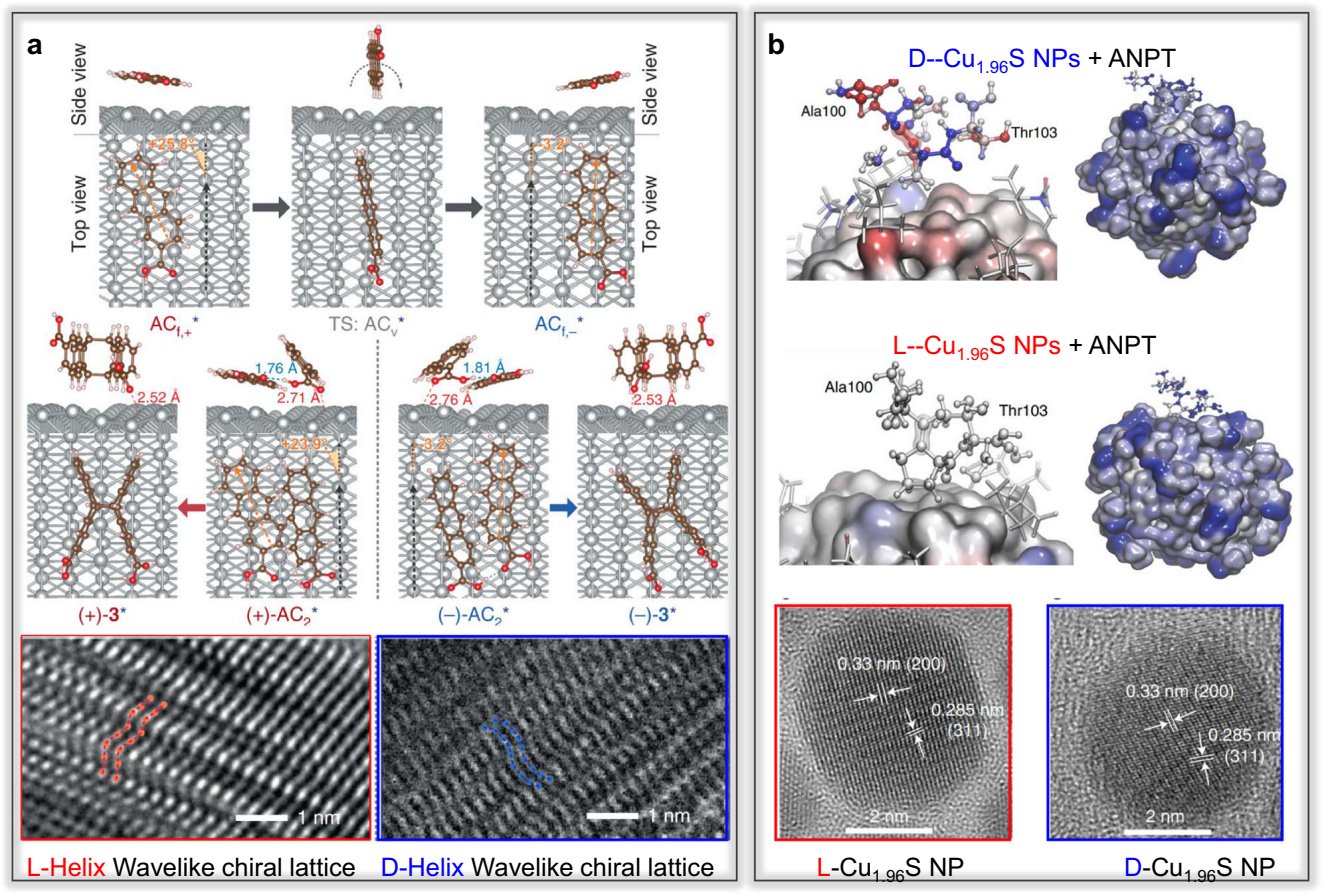
Computer simulation of enantioselective catalysis mediated by chiral inorganic nanocatalysts. **a** Density functional theory calculation of the helicity-dependent photochirogenic cyclodimerization of AC^61^. **b** Molecular dynamic simulation of polarization maps for the Ala 100–Asn 101–Pro 102–Thr 103 fragment (ANPT) proximal to the surface of the chiral NPs, and molecular organization of the L-/D-NP–peptide complex for the ANPT fragment^76^.

Furthermore, chiral metal nanocatalysts can be constructed without using chiral molecules as selectors or inductors. Zhang et al.^61^ demonstrated this by controlling the rotating deposition angle onto a substrate to create Ag or Cu nanohelices. These nanohelices facilitated the absorption of 2-Anthracenecarboxylic (AC) molecules on their surfaces, resulting in the formation of enantiomorphous anti-head-to-head dimers with Si-Si or Re-Re facial stacking, depending on the handedness of the nanohelices. Specifically, nanohelices with left-handedness facilitated the synthesis of (+)-cyclodimers through enantioselective photocatalysis, whereas those with right-handedness promoted the formation of (-)-cyclodimers (Fig. 1a, Fig. 3a). In addition, the potential of neutrophil membrane-coated chiral Pd catalysts in mediating chiral catalysis for the biorthogonal synthesis of ibuprofen was discovered, showing promise in alleviating in vivo inflammation^62^. This work introduced the concept of localized prodrug activation synthesis mediated by chiral catalysts, opening up new possibilities in this area.

### Chiral metal oxide nanocatalysts

Chiral metal oxide nanocatalysts have been instrumental in facilitating the synthesis of chiral chemicals through electrochemical and redox reactions. These catalysts are typically synthesized using the sol-gel method, hydrothermal method, and precipitation method. These kinds of catalysts are known for their active, selective, and energy-efficient catalytic performances, as well as their abundance in Earth’s resources^63–66^. In the initial stages of chiral metal oxide nanocatalysts development, metal oxide NPs were commonly employed to support chiral organic catalysts. This approach aimed to improve the recyclability and reusability of chiral organic catalysts. For example, Hosseini-Monfared et al. loaded manganese-porphyrin, which contained (1 R, 2 S)-(+)-cis-1-amino-2-indanol, onto the surface of Fe_3_O_4_ NPs^42^.

Later, it is found that metal oxide nanomaterials can also act as catalytic centers due to their redox and electrochemical catalytic ability. As an example, poly(amino acid)-coated Fe_3_O_4_ NPs were employed to facilitate the dimerization of L-/D-tyrosinol with significant enantioselectivity^67^. Here, Fe_3_O_4_ functioned as the active catalytic centers, while poly(amino acid)s served as chiral selectors. The selectivity factor reached a remarkable value of 5.38. Theoretical calculations revealed that the adsorption-free energy of D-tyrosinol (L-tyrosinol) on the outer surface of Poly(D-Trp) was significantly lower than that of L-tyrosinol, indicating that Poly(D-Trp) exhibited a higher affinity for D-tyrosinol compared to L-tyrosinol, and vice versa. It is worth noting that the application of targeted catalytic processes has been extended to bacterial cells, thereby successfully expanding the application scope of chiral catalysts.

Other metal oxide NPs, such as cerium oxide (CeO_2_) and tungsten trioxide (WO_3-x_), also show considerable potential for application as chiral catalysts. Amino acids play crucial roles in controlling the stereochemistry and stereoselectivity of chiral metal nanocatalysts. Kotov’s team demonstrated that WO_3-x_ NPs could be synthesized with strong chiral optical activities in the near-infrared range by employing proline (Pro) and aspartic acid (Asp) as chiral ligands^47^. The presence of chiral molecules on the mineral surface contributes to the catalytic ability of WO_3-x_ NPs, facilitating the formation of peptide bonds involving Asp-Asp and Asp-Pro. Qu and colleagues^68^ explored the modification of ceria oxide (CeO_2_) NPs using various amino acids. They found that phenylalanine (Pen)-modified ceria oxide NPs showed the highest stereoselectivity towards the enantiomers of DOPA compared to NPs modified with other amino acids. Furthermore, *L*-Pen-modified ceria oxide NPs showed higher catalytic oxidation towards D-DOPA, whereas *D*-Pen-modified ceria oxide NPs demonstrated higher catalytic oxidation towards *L*-DOPA. These studies highlight the potential of grafting chiral molecules onto inorganic nanocatalysts to achieve stereoselectivity. Additionally, ZnO, CuO, Fe_2_O_3_, Co_3_O_4_, and WO_3_, in conjunction with Macmillan’s chiral secondary amine catalyst, have been used as photocatalysts. Among these nanomaterials, WO_3_ exhibited superior yield and enantiomeric excess (ee) due to its suitable bandgap (2.6 eV) for visible light absorption, oxidation of alpha-radical intermediates, and reduction of C-Br. This approach offers a practical strategy for developing photocatalysts for asymmetric reactions^69^. Despite emerging research on chiral metal oxide nanocatalysts, their development remains in its infancy due to the demanding requirements of innovative materials design.

### Chiral semiconductor nanocatalysts

Photosynthetic bacteria are recognized as the earliest and most prevalent organisms capable of harnessing light energy to produce organic molecules. Additionally, semiconductor nanomaterials with suitable band gaps and specific surfaces are versatile materials capable of catalyzing photocatalytic reactions by harnessing photoinduced electrons and holes^70–73^. The question of whether semiconductor materials can mimic natural enzymes by exhibiting pronounced stereoselectivity towards chiral chemicals through photon energy has garnered significant interest^33,70,74,75^. Chiral semiconductor materials are usually synthesized directly or through post-modification methods involving chiral molecules. The direct synthetic method encompasses microwave-induced heating, aqueous synthetic method, and microwave irradiation. Post-modification methods typically involve ligand exchange using chiral molecules and the coassembly of semiconductor nanocrystals with chiral supramolecular gelators. Both direct synthesis and ligand exchange with chiral molecules are commonly utilized approaches for constructing chiral semiconductor nanocatalysts.

Kuang et al.^36^ showed that chiral Cys-modified CdTe NPs could specifically recognize the GATATC restriction site within double-stranded DNA sequences. When exposed to light, these DNA sequences were cleaved precisely at the GAT and ATC positions. This specific recognition was attributed to the chiral affinity between Cys and the designated site within the DNA sequence. The excitation of photons resulted in generating reactive oxygen species and led to the cleavage of the DNA strands. Importantly, this study demonstrated that Cys-modified CdTe NPs could be utilized for DNA cleavage both within living cells and in vivo (Fig. 1c). These findings established that inorganic nanomaterials could be engineered with specific chiral configurations to emulate the bifunctional characteristics of nucleases.

A recent discovery by the same authors demonstrated that chiral Cu_1.96_S NPs effectively cleaved capsids present in the tobacco mosaic virus with site selection under sunlight. This effectiveness can be attributed to the strong affinity between D-Pen stabilized Cu_1.96_S NPs and a specific segment (Gln99 to Ala105) within the capsids^76^. This study established the potential use of chiral NPs as antiviral agents. Molecular dynamic simulations revealed that Cu_1.96_S NPs could readily penetrate the TMV capsid cavity and interact with the capsid protein monomers through supramolecular bonding, with a significant electrostatic component (Fig. 3b).

Furthermore, Pen-modified Cu_2-x_S quantum dots (QDs) were discovered to exhibit high enantioselectivity in the degradation of bovine serum albumin (BSA) due to the generation of hydroxyl radicals and the chiral preference between Pen and BSA^77^. Chiral Fe_x_Cu_y_Se NPs, stabilized with L-/D-Pen, displayed strong chiral signals from the UV to near-infrared range^71^. These NPs effectively inhibited the aggregation of Aβ42 monomers and even dissociated Aβ42 fibrils under 808 nm near-infrared illumination. Notably, *D*-Pen-modified Fe_x_Cu_y_Se NPs exhibited superior inhibition and dissociation capabilities relative to their *L*-Pen-modified counterparts, both in vitro and in vivo, owing to the augmented affinity between *D*-Pen and Aβ42. This discovery holds significant promise in the prevention of Alzheimer’s disease pathology.

In a study by Xu et al.^49^, a series of chiral CdSe, CdS, CdSe@CdS, CdS-Au, and CdS-Pt semiconductor nanorods were synthesized using Cys as surface chiral ligands. These semiconductor nanorods, when combined with metal deposits, exhibited remarkable photocatalytic capabilities due to enhanced energy transfer and improved separation of photoinduced electrons and holes. Recently, Wei et al.^72^ utilized MoS_2_ and WS_2_ as inorganic cores to construct chiral transition-metal dichalcogenide quantum dots (QDs), which displayed excellent peroxidase-like catalytic performance with high chiral selectivity in the presence of Cu^2+^. The enantioselectivity of these chiral QDs towards chiral substrates (*L*-/*D*-tyrosinol) reached an impressive value of 6.77.

Although inorganic nanocatalysts exhibit impressive enantioselective catalytic capabilities towards chiral substrates, their effectiveness is significantly hindered by the dimensional mismatch between the geometries and the inorganic nanomaterials. To address these challenges, self-assembled ZnS supraparticles were introduced, achieving a balance between short-range attraction and long-range repulsion among individual ZnS NPs^37,38^. This assembly process resulted in the formation of numerous chiral three-dimensional recognition spaces within the ZnS supraparticles, ranging in size from the sub-nanoscale to nanoscale (Fig. 1d). The presence of these three-dimensional chiral spaces greatly enhanced both the enantioselectivity and catalytic efficiency compared to individual ZnS NPs. Moreover, the composition of the supraparticles could be flexibly regulated to meet the specific requirements of various catalytic reactions. This groundbreaking research has opened up a new avenue for constructing chiral catalysts with precise functionalities akin to biological nanoassemblies.

### Chiral composite inorganic nanomaterials

Combining nanocomposite materials with different constituents allows for the integration of diverse functional properties and imparts enhanced catalytic capabilities to the nanomaterials^78–83^. However, the chiral composite nanocatalysts still need to be improved due to the high complexity of the material construction and the challenges in precisely controlling catalysis-related properties. A notable example of chiral composite nanocatalysts is the controlled assembly of gold (Au) and titanium dioxide (TiO_2_) NPs on the chiral template of silicon dioxide (SiO_2_) nanoribbons, leading to the formation of polarization-dependent photocatalysts^51^. By exploiting electromagnetic excitation, this assembly generates asymmetric distributions of hot electrons and holes, facilitating asymmetric photocatalysis under plasmonic excitation.

In another study by Ma et al., a series of chiral CdSe@CdS semiconductor nanorods were developed, and their photocatalytic activities were significantly enhanced through post-growth modifications with platinum (Pt) or gold (Au) at multiple sites. This work offers a straightforward approach to producing asymmetric photocatalysts by adjusting the morphology and composition of semiconductor nanorods^49^.

## Challenges in the field

The controllability of stereochemistry, the size effect, and specificity are critical parameters for chiral inorganic nanocatalysts, as they play indispensable roles in governing catalytic processes. However, all these aspects become challenging when dealing with inorganic nanomaterials due to the complexities associated with precisely regulating and controlling their structural, chemical, and catalytic properties. The scientific community must address these limitations in the years to come. In this section, we will highlight some of these specific challenges and propose viable solutions to overcome them^8^.

Firstly, the stereochemical control in chiral inorganic nanocatalysts towards chemical substrates is limited, resulting in low enantioselectivity or specificity. The stereoselectivity of chiral inorganic nanocatalysts is mainly related to the size and configuration of the chiral recognition center. Enhancing stereoselectivity can be achieved by modifying recognition molecules with chiral cavities on the surface of inorganic materials, including crown ethers, calixarenes, and cyclodextrins^84^. An alternative method involves directly synthesizing inorganic nanocatalysts with specific chiral pattern arrangements^54^, which can also enhance chiral selectivity. Secondly, the significant dimensional mismatch between chiral inorganic nanocatalysts and catalytic substrates poses a major obstacle to achieving enantioselective catalytic activities. Creating confined spaces that match the dimensional size of catalytic substrates in chiral inorganic nanocatalysts is the most effective way to solve the dimensional mismatch. Various construction methods can be explored to create chiral inorganic nanocatalysts with finite chiral recognition space. These methods include self-assembly, which combines chiral NPs to form chiral internal cavities^37,85^, selective etching of chiral molecules embedded in inorganic nanomaterials^52^, or modifying chiral polymer molecules on the surface of inorganic nanocatalysts^67^. Thirdly, different catalytic reactions require distinct redox properties. Consequently, inorganic nanocrystals serving as active catalytic sites should possess diverse chemical, electrical, and optical properties to meet various catalytic demands. Exploring general construction methods capable of regulating the inorganic composition to align with the redox requirements of different catalytic reactions should be feasible. Combining different kinds of materials in an orderly manner using the self-assembly method would be effective due to its high flexibility in regulating the building blocks^37^. Fourthly, in many chiral inorganic nanomaterials, only the surface portion acts as the catalytic site and participates in catalytic processes, leading to low utilization efficiency. It is crucial to ensure the involvement of internal crystals in the catalytic processes and improve substrate diffusion efficiency. Developing chiral inorganic nanocatalysts with numerous internal pores holds the potential to overcome this difficulty^53,59^. Fifth, exploring systematic calculation and simulation methods to guide the design of chiral inorganic nanocatalysts and predict the possible outcomes is also a significant challenge^26,86,87^. Strong theoretical components are essential to understand better the relationship between the multiscale chirality of nanostructures and potential applications in enantioselective catalysis. Developing theoretical aspects on the chirality of inorganic nanomaterials^88,89^ is essential to enable the facile implementation of artificial intelligence and data science methods for their property-optimized synthesis of chiral inorganic nanocatalysts. Density functional theory-based and molecular dynamic calculations are often used to explain extraordinary experimental phenomena in enantioselective catalysis (Fig. 3). Finally, the catalytic mechanisms and the details of many catalytic reactions remain ambiguous. Further research should elucidate the comprehensive mechanisms behind the catalytic processes.

## Conclusions and prospects

By understanding the relationship between the structure and function of chiral inorganic nanomaterials, we can address the challenges and limitations faced in chiral catalysis. Moving forward, we expect that research will lead to the development of unique morphologies, enhanced solvent compatibility, cost-effectiveness, and superior bio-mimetic catalytic performances in chiral inorganic nanocatalysts. These advancements will have positive impacts on various fields, such as biological systems, agriculture, biomedical science, and environmental science, extending beyond enantioselective organic synthesis. The advancements in this area hold the potential to redefine catalysis and revolutionize scientific thinking in the coming decade.
